# Supplementary material for: The global RNA-binding protein RbpB is a regulator of polysaccharide utilization in Bacteroides thetaiotaomicron
Source: Nat Commun. 2025 Jan 2;16:208. doi: 10.1038/s41467-024-55383-8 (PMC11697453; doi:10.1038/s41467-024-55383-8)
Supplement: Supplementary file 1 — Supplementary Information [file 41467_2024_55383_MOESM1_ESM.pdf]

SUPPLEMENTARY INFORMATION

The global RNA-binding protein RbpB is a regulator of polysaccharide utilization in *Bacteroides thetaiotaomicron*

Ann-Sophie Rüttiger[1,2], Daniel Ryan[1,2], Luisella Spiga[3], Vanessa Lamm-Schmidt[2,4], Gianluca Prezza[2], Sarah Reichardt[2], Madison Langford[3], Lars Barquist[2,5,6], Franziska Faber[2,7], Wenhan Zhu[3], Alexander J. Westermann[1,2*]

[1]Department of Microbiology, Biocenter, University of Würzburg, Würzburg, D-97074, Germany

[2]Helmholtz Institute for RNA-based Infection Research (HIRI), Helmholtz Centre for Infection Research (HZI), Würzburg, D-97080, Germany

[3]Department of Pathology, Microbiology, and Immunology, Vanderbilt University, Nashville, Tennessee, USA

[4]Institute of Molecular Infection Biology, University of Würzburg, Würzburg, D-97080, Germany

[5]Faculty of Medicine, University of Würzburg, Würzburg, D-97080, Germany

[6]Department of Biology, University of Toronto, Mississauga, L5L 1C6, Ontario, Canada

[7]Institute for Hygiene and Microbiology, University of Würzburg, Würzburg, D-97080, Germany

[*]Correspondence: alexander.westermann@uni-wuerzburg.de

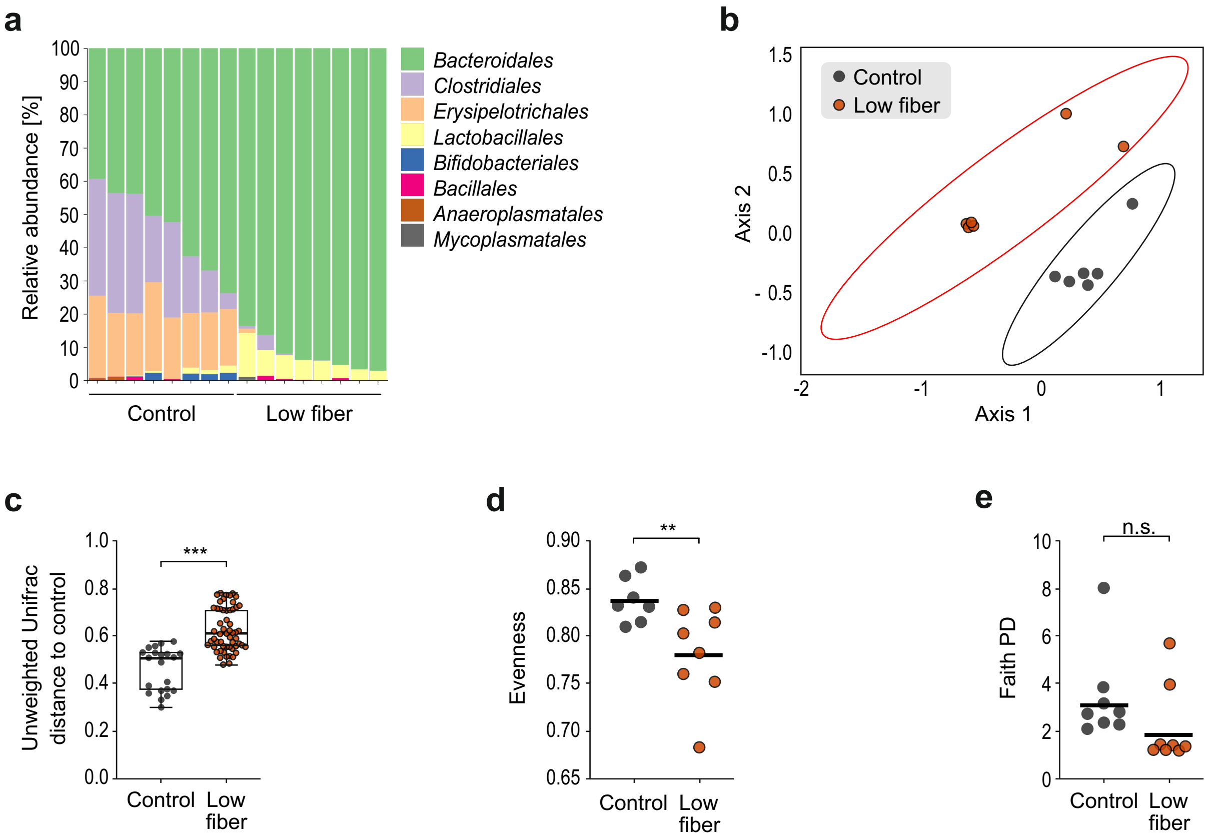

Supplementary Figure 1: 16S profiling. a-e, C57BL/6J mice were switched to a low fiber diet or remained on the fiber-rich control diet until the end of the experiment. Antibiotic cocktails were administered by oral gavage daily for 5 days. Mice were inoculated with an equal mixture of 0.5 x 10^9 CFU of the *B. thetaiotaomicron* wild-type and the *rbpB* mutant strains. Colonic contents were collected six days post inoculation and their DNA extracted. 16S rRNA gene sequencing was performed to assess (**a**) taxonomy composition; (**b, c**) β-diversity: principle coordinate analysis (**b**), unweighted unique fraction metric (**c**); and (**d, e**) α-diversity: evenness (**d**), Faith's phylogenetic diversity (**e**).

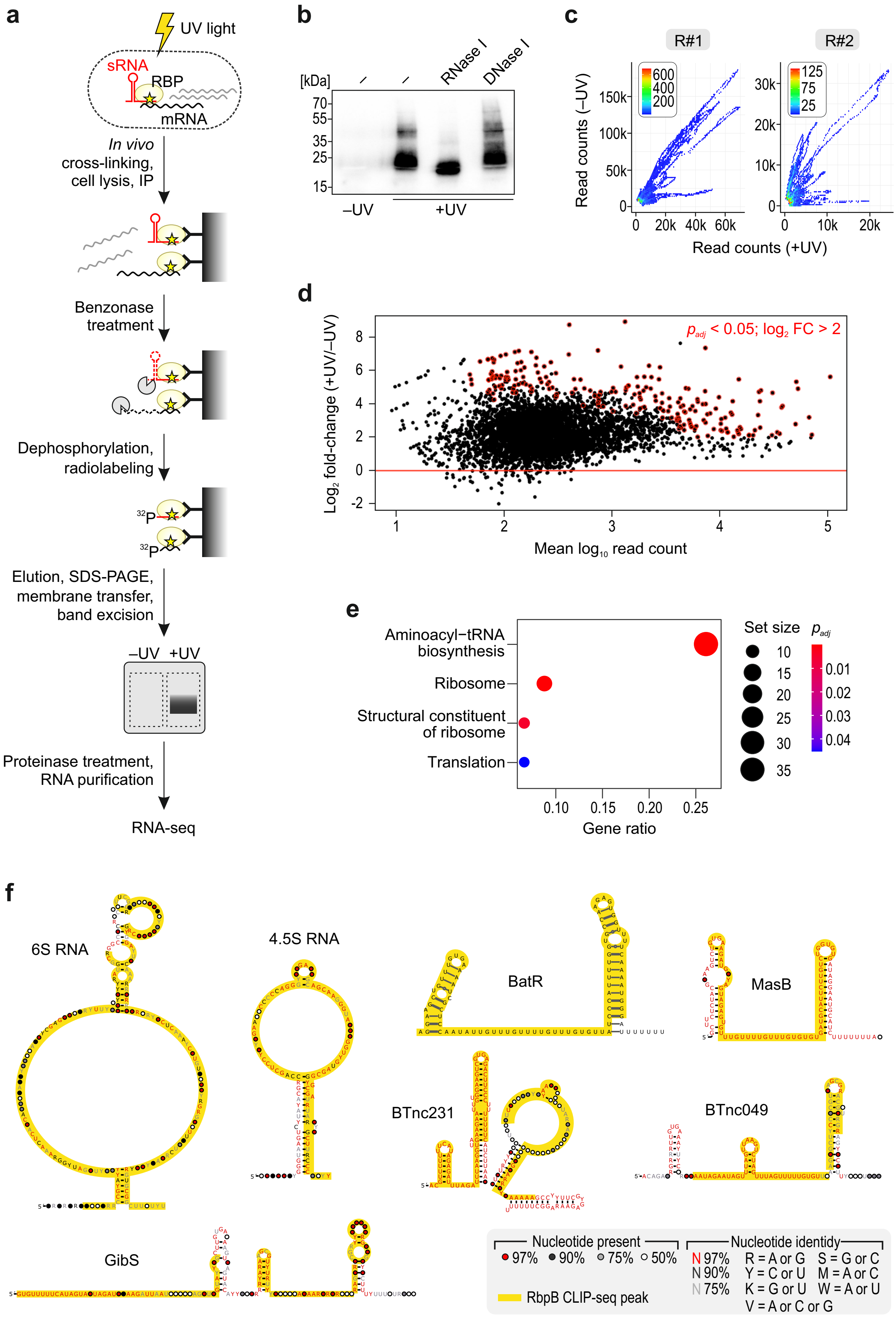
a
UV light
sRNA
RBP
mRNA
In vivo cross-linking, cell lysis, IP
Benzonase treatment
Dephosphorylation, radiolabeling
Elution, SDS-PAGE, membrane transfer, band excision
–UV +UV
Proteinase treatment, RNA purification
RNA-seq
b
[kDa]
RNase I
DNase I
70
55
35
25
15
–UV
+UV
c
R#1
R#2
Read counts (–UV)
Read counts (+UV)
d
Log2 fold-change (+UV/–UV)
Mean log10 read count
padj < 0.05; log2 FC > 2
e
Aminoacyl−tRNA biosynthesis
Ribosome
Structural constituent of ribosome
Translation
Gene ratio
Set size
padj
f
6S RNA
4.5S RNA
BatR
MasB
BTnc231
BTnc049
GibS
Nucleotide present
97% 90% 75% 50%
RbpB CLIP-seq peak
Nucleotide identidy
N 97%
N 90%
N 75%
R = A or G
Y = C or U
K = G or U
V = A or C or G
S = G or C
M = A or C
W = A or U

Supplementary Figure 2: RbpB CLIP-seq. a, Schematic workflow of the CLIP-seq procedure to identify the interactome of bacterial RBPs. Cells are irradiated with UV light (254 nm) to covalently bind protein and RNA (yellow asterisk). Upon lysis and immunoprecipitation using antibodies against the FLAG-epitope, RNP complexes are partially degraded by benzonase and subjected to radioactive labeling with polynucleotide kinase. RNPs are separated via SDS-PAGE, transferred to a nitrocellulose membrane, and the radioactive section of the membrane as well as the corresponding area in the non-crosslinked control are excised. Protein-bound RNA is released from the membrane upon proteinase K digestion and further purified through phenol:chloroform:isoamyl alcohol extraction. For RNA-seq, adapters are ligated to the RNA fragments followed by reverse transcription of RNA into cDNA, and PCR amplification. **b,** Testing the sensitivity of RbpB co-purifications to RNase I and DNase I. Shown is the autoradiogram of a CLIP assay performed with samples treated with the indicated nucleases. **c,** Frequency plots of matched crosslinked and background samples for the two independently performed experiments. Plotted are the read counts per genetic feature in the crosslinked (x-axes) and non-crosslinked (y-axes) samples and the coloring refers to the frequency of each x–y pair. **d,** MA-plot showing $\log_2$ fold-changes for identified peaks between crosslinked libraries and non-crosslinked control libraries. Peaks defined as enriched across both crosslinked libraries, with an adjusted p value < 0.05 and $\log_2$ fold-change > 2, are highlighted in red. **e,** Pathway enrichment analysis of RbpB-bound mRNAs. Genes with at least one significant CLIP peak were subjected to a functional enrichment analysis based on a custom annotation (see Methods section). Set size indicates the number of genes in the gene set that contain a CLIP peak and gene ratio is defined as the set size divided by the total number of genes with CLIP peaks. **f,** RbpB binding sites within noncoding RNAs for which the secondary structure is known, namely the 4.5S and 6S housekeeping RNAs, the known *trans*-acting sRNAs GibS, MasB, BatR, and the regulatory sRNA candidates BTnc049 and BTnc231. Secondary structure information from [1,2]. Source data are provided as a Source Data file.

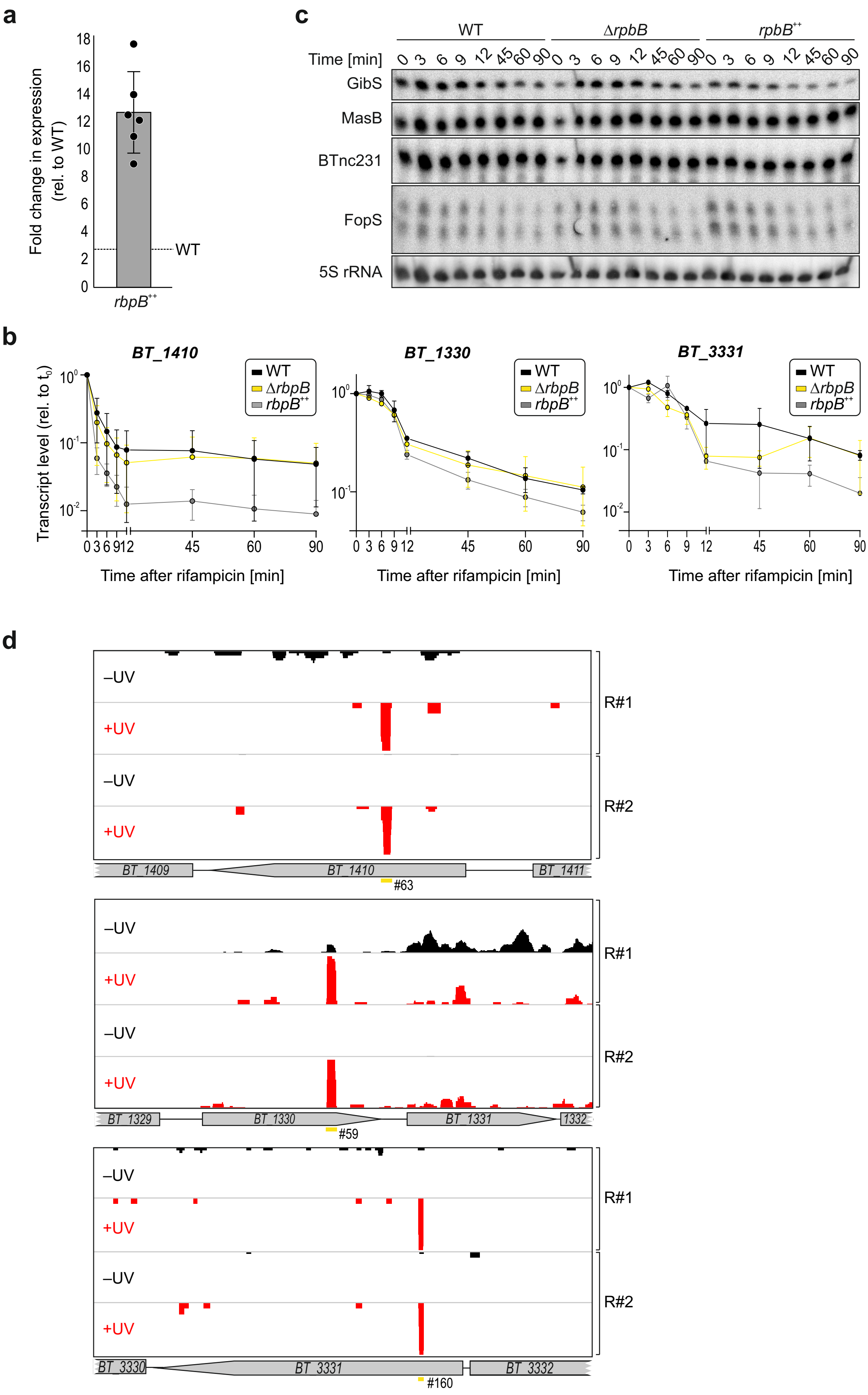

a
Fold change in expression (rel. to WT)
18
16
14
12
10
8
6
4
2
0
WT
rbpB++
c
WT
ΔrpbB
rpbB++
Time [min]
0 3 6 9 12 45 60 90
GibS
MasB
BTnc231
FopS
5S rRNA
b
BT_1410
BT_1330
BT_3331
WT
ΔrbpB
rbpB++
Transcript level (rel. to t0)
Time after rifampicin [min]
d
–UV
+UV
R#1
R#2
BT_1409
BT_1410
BT_1411
#63
BT_1329
BT_1330
BT_1331
1332
#59
BT_3330
BT_3331
BT_3332
#160

Supplementary Figure 3: Cellular half-lives of RNA ligands of RbpB. a, Characterization of the *rbpB* overexpression strain (*rbpB*$^{++}$) used in this study. Plotted is the fold-change in expression of the *rbpB* mRNA in *rbpB*$^{++}$ *B. thetaiotaomicron* relative to its level in the isogenic wild-type grown in TYG to mid-exponential phase, as determined by qRT-PCR measurement and normalization against the 16S rRNA transcript (six biological replicates). Note, however, that strain *rbpB*$^{++}$ expresses a C-terminally FLAG-tagged variant of RbpB (as was used for CLIP). **b, c,** Rifampicin-chase experiments to determine the stability of RbpB-associated RNA ligands. Total RNA was collected from wild-type, Δ*rbpB*, and *rbpB*$^{++}$ *B. thetaiotaomicron* cultures grown in TYG to mid-exponential phase prior to rifampicin treatment (final concentration: 500 µg/mL) for the indicated time periods. The purified RNA samples were DNase-digested and used for qRT-PCR-based quantification of the decay kinetics of selected mRNA ligands of RbpB (top three mRNA interactors based on the enrichment score compared to the non-crosslinked control), normalized against 16S rRNA levels (**b**; plotted are the means +/-SD from three independent replicate measurements). Alternatively, RNA samples were loaded on a northern blot and probed with sequence-specific, radioactively labeled oligonucleotides against established sRNAs [3,4] that were significantly enriched in the RbpB CLIP-seq data, or a probe for 5S rRNA as a non-enriched control (**c**; blots are representative of three biological replicates). Note that endogenous *rbpB* is only lowly expressed in wild-type *B. thetaiotaomicron* grown in TYG medium [3] , which might explain the similarities between wild-type and Δ*rbpB* bacteria in these assays. **d,** Relative position of RbpB CLIP-seq peaks (peak ID marked in yellow) within the mRNA ligands, whose stability was quantified in panel **b**. Source data are provided as a Source Data file.

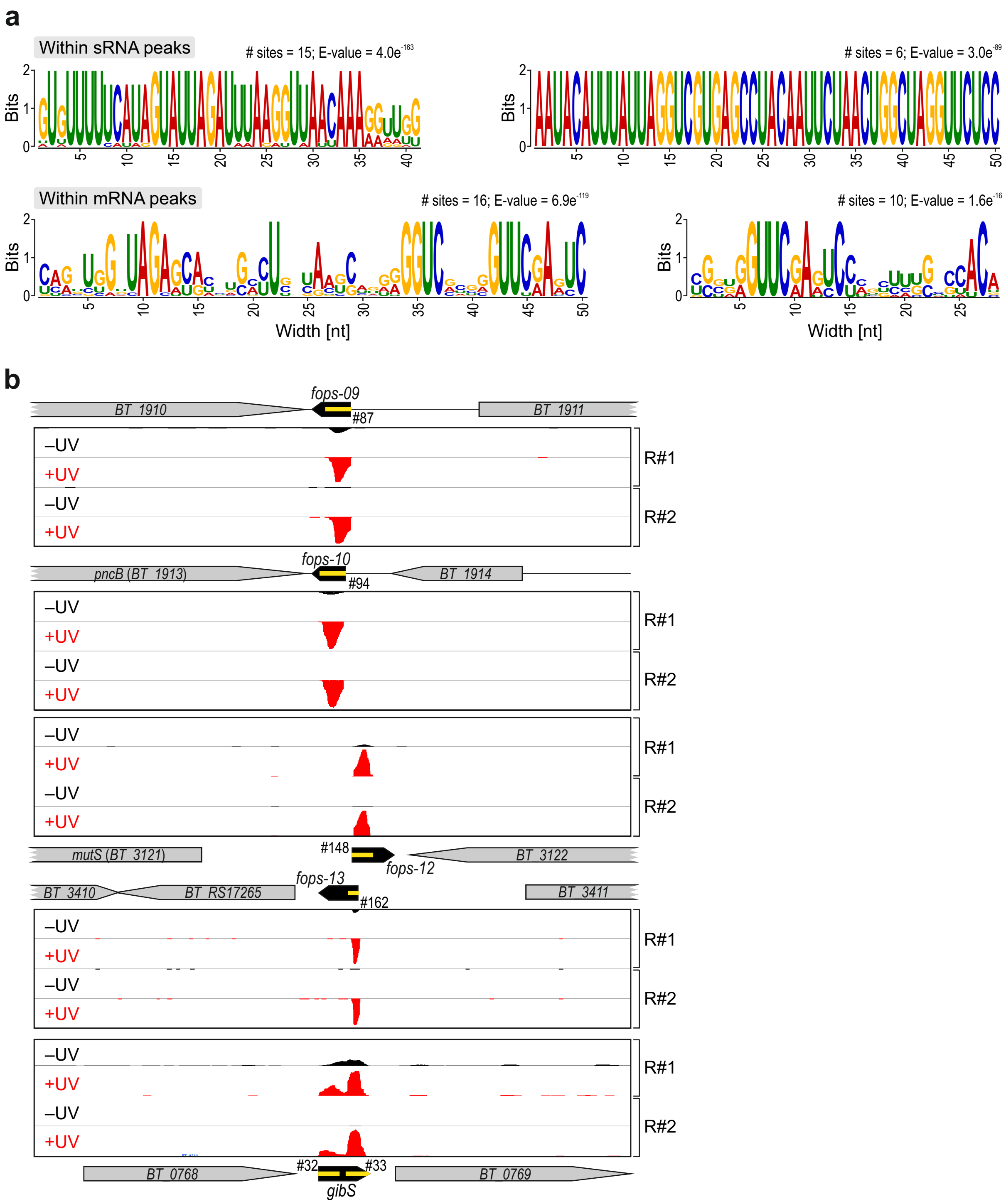

Supplementary Figure 4: RpbB binds to specific sequence motifs. a, Most over-represented sequence motifs within RbpB peak regions. MEME results for the top-enriched motifs in sRNAs (upper) and mRNAs (lower); their prevalence and E-values are given. **b,** Representative CLIP peaks within sRNAs harboring the 41-nt motif depicted in panel **a** (upper left). Shown are the scale-matched read coverages in control (black) and crosslinked (red) libraries. Annotations of coding sequences (grey arrows) and sRNA genes (black arrows) are given. Yellow horizontal lines indicate the inferred peak positions (peak IDs are given).

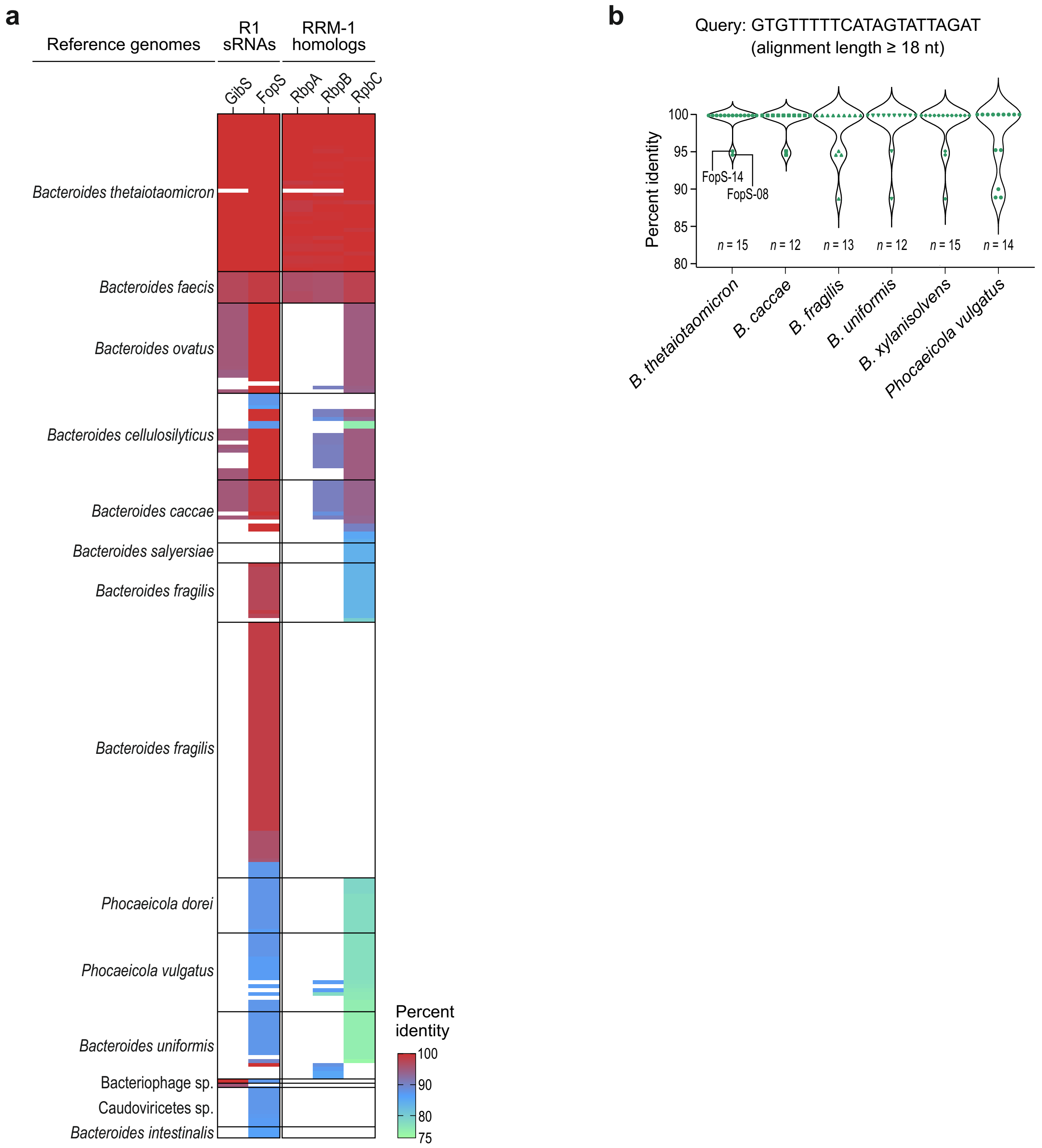

Supplementary Figure 5: Conservation and prevalence of R1 sequence-containing sRNAs and of homologs of RRM-1 proteins across the *Bacteroidaceae*. a, BLAST analysis of GibS, the FopS family, and the three RRM-1 proteins RbpA, -B, and -C. In case of the FopS cluster, we blasted for FopS-08, which—based on CD-HIT clustering[5], with 65% sequence identity—is most representative for this sRNA family. The alignments were performed at default settings, except with increasing the maximum number of aligned sequences to 250. The heat map is color-coded based on sequence identity ranging from 75% (green) up to 100% (red). The Caudovirales phage was predicted to target *Bacteroides* based on matching CRISPR spacers[6]. **b,** Prevalence of R1 sequence copies within prominent *Bacteroidaceae* genomes, as inferred from a BLAST analysis.

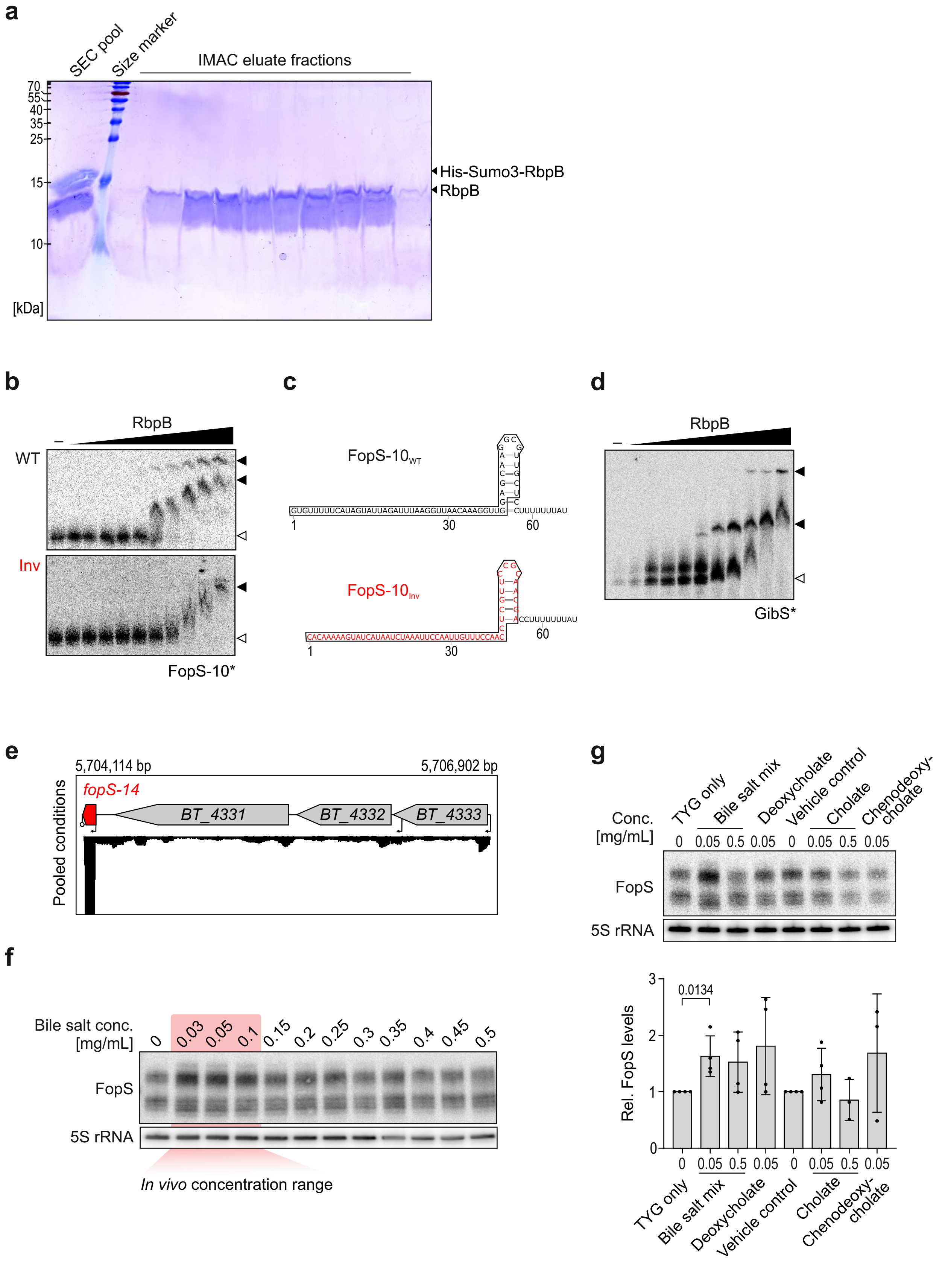

a
SEC pool
Size marker
IMAC eluate fractions
70
55
40
35
25
15
10
[kDa]
His-Sumo3-RbpB
RbpB
b
RbpB
WT
Inv
FopS-10*
c
FopS-10WT
FopS-10Inv
d
RbpB
GibS*
e
5,704,114 bp
5,706,902 bp
fopS-14
BT_4331
BT_4332
BT_4333
Pooled conditions
f
Bile salt conc. [mg/mL]
0 0.03 0.05 0.1 0.15 0.2 0.25 0.3 0.35 0.4 0.45 0.5
FopS
5S rRNA
In vivo concentration range
g
Conc. [mg/mL]
TYG only
Bile salt mix
Deoxycholate
Vehicle control
Cholate
Chenodeoxy-cholate
0 0.05 0.5 0.05 0 0.05 0.5 0.05
FopS
5S rRNA
Rel. FopS levels
0.0134

Supplementary Figure 6: *In-vitro* validation of RbpB–sRNA interactions and expression profiling of FopS sRNAs. a, Size-exclusion chromatography (SEC) purification of recombinant His-Sumo3-RbpB expressed in *E. coli* Bl21. The soluble lysate was subjected to immobilized metal affinity chromatography (IMAC) and analyzed by SDS-PAGE. The tagged protein has a molecular weight of 23.4 kDa (RbpB itself has 11.0 kDa). **b,** FopS-10 EMSAs. Increasing concentrations of purified RbpB (up to 80 µM) were incubated with either wild-type T7-transcribed and 5' end-labeled FopS-10 or a mutated variant thereof (4 nM). White and black arrows refer to free and bound FopS-10, respectively. **c,** Mutation of the RbpB binding site within FopS-10. Depicted is the sequence and predicted secondary structure of FopS-10 variants: wild-type (black) and inverted (red). The 55 nt-long RbpB-binding site is boxed. **d,** *In vitro*-transcribed and 5' end-labeled GibS (4 nM) was incubated with increasing concentrations of RbpB (80 µM maximum). White and black arrows refer to free and bound GibS, respectively. **e,** FopS-14 is a 3'-derived sRNA. The combined read coverages from Theta-Base [4] across the *fopS-14* locus is shown. **f, g,** Northern blot analysis of FopS sRNA expression across a bile salt concentration range, (0–0.5 mg/mL) (**f**) and upon exposure to the defined bile salts deoxycholate, cholate, and chenodeoxycholate, in the indicated concentrations (**g**). The bile salt mix consisted of cholic acid (a primary bile salt) and deoxycholate (a conjugated, secondary bile salt) in equimolar ratio. The bar graph in panel **g** shows the mean FopS levels normalized to 5S rRNA and error bars indicate the SD over three to four independent replicate experiments, plotted as individual dots. Significance was assessed using an unpaired *t*-test. Source data are provided as a Source Data file.

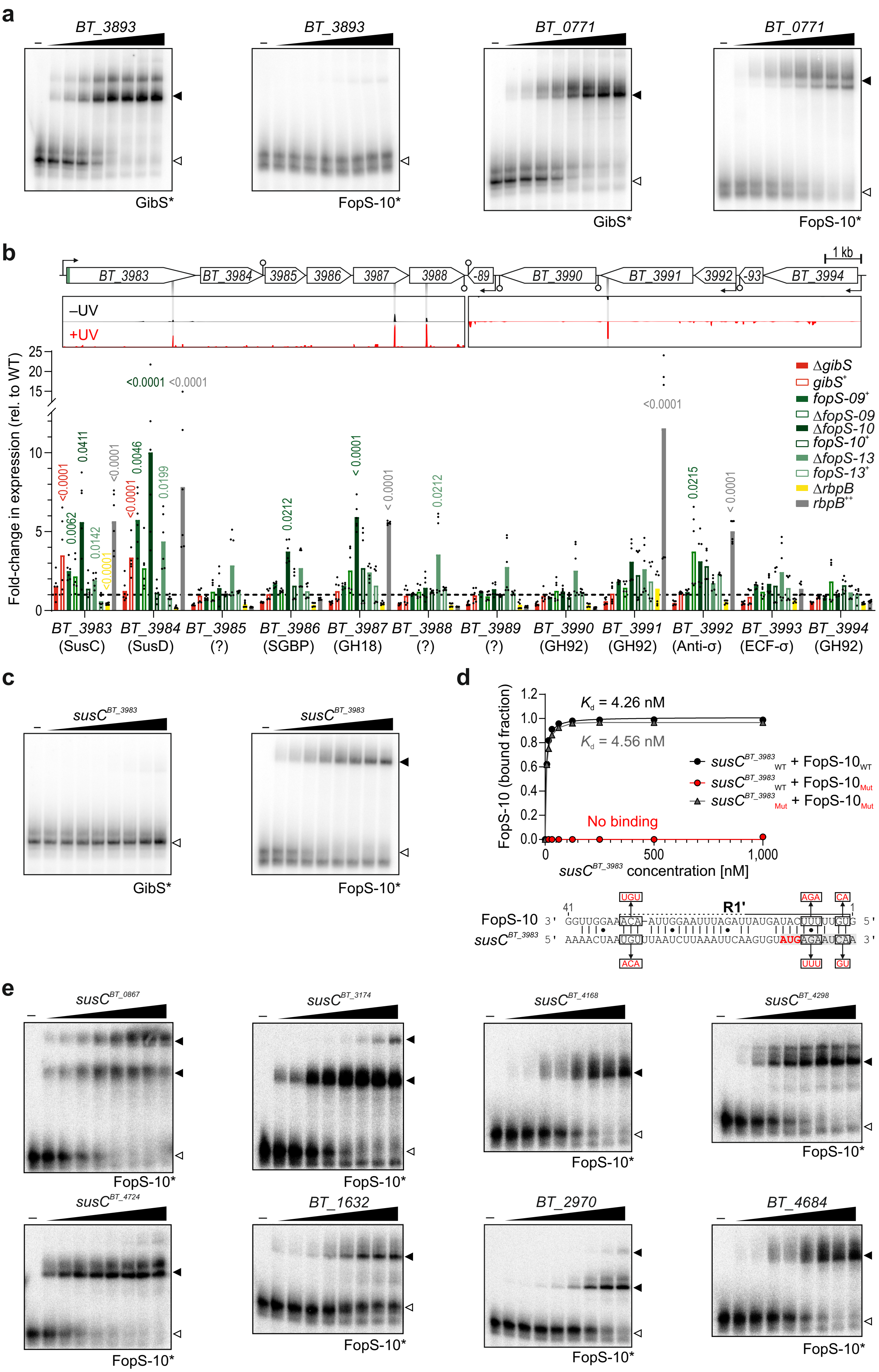
a
BT_3893
BT_3893
BT_0771
BT_0771
GibS*
FopS-10*
GibS*
FopS-10*
b
BT_3983
BT_3984
3985
3986
3987
3988
-89
BT_3990
BT_3991
3992
-93
BT_3994
1 kb
–UV
+UV
Fold-change in expression (rel. to WT)
ΔgibS
gibS+
fopS-09+
ΔfopS-09
ΔfopS-10
fopS-10+
ΔfopS-13
fopS-13+
ΔrbpB
rbpB++
BT_3983 (SusC)
BT_3984 (SusD)
BT_3985 (?)
BT_3986 (SGBP)
BT_3987 (GH18)
BT_3988 (?)
BT_3989 (?)
BT_3990 (GH92)
BT_3991 (GH92)
BT_3992 (Anti-σ)
BT_3993 (ECF-σ)
BT_3994 (GH92)
c
susC BT_3983
susC BT_3983
GibS*
FopS-10*
d
Kd = 4.26 nM
Kd = 4.56 nM
No binding
FopS-10 (bound fraction)
susC BT_3983 concentration [nM]
susC BT_3983 WT + FopS-10 WT
susC BT_3983 WT + FopS-10 Mut
susC BT_3983 Mut + FopS-10 Mut
R1'
FopS-10 3' GGUUGGAAACA-AUUGGAAUUUAGAUUAUGAUACUUUUUGUG 5'
susC BT_3983 5' AAAACUAAUGUUUAAUCUUAAAUUCAAGUGUAUGAGAAUCAA 3'
UGU
AGA
CA
ACA
UUU
GU
e
susC BT_0867
susC BT_3174
susC BT_4168
susC BT_4298
susC BT_4724
BT_1632
BT_2970
BT_4684
FopS-10*

Supplementary Figure 7: FopS target identification and validation. a, EMSAs with established GibS targets. *In vitro*-transcribed and 5' end-labeled GibS or FopS-10 (4 nM each) was incubated with increasing concentrations (up to 1 mM) of ~150 nt long 5' segments of either *BT_3893* or *BT_0771*. White or black arrows refer to free or bound GibS and FopS-10, respectively. **b,** qRT-PCR measurement of the steady-state levels of PUL72-derived transcripts. Indicated strains were grown in TYG for 7 h and exposed to 0.5 mg/mL of bile salts for 2 h prior to RNA extraction and analysis. Significance was assessed using two-way ANOVA (Sidak's multiple comparisons test; only significant *p* values [$p < 0.05$] are indicated). The genomic locus of PUL72 is represented at the top, with the FopS binding site (see Fig. 4e) in green and putative RbpB binding sites (as manually inferred from CLIP-seq [note that these are not significant according to our default cutoffs; read coverages from one representative CLIP-seq replicate at the top]; which might be responsible for some of the altered transcript levels in the $rbpB^{++}$ mutant) in grey. The data for *BT_3983* and *BT_3984* are the same as those plotted in Fig. 4c. **c-e,** EMSAs to confirm FopS-10 binding to the predicted target mRNA $susC^{BT\_3983}$ (**c**, **d**) and to the mRNAs encoding SusC homologs from other PULs ($susC^{BT\_0867}$ [PUL12; substrate: mucin *O*-glycans [7]], $susC^{BT\_3174}$ [PUL51; substrate unknown], $susC^{BT\_4168}$ [PUL77; substrate: rhamnogalacturonan I [8,9]], $susC^{BT\_4298}$ [PUL80; substrate: host glycans, likely mucin *O*-glycans [7]], $susC^{BT\_4724}$ [PUL88; substrate unknown]), of a putative chitinase of PUL20 (*BT_1632*; substrate: host glycans of unknown type [7]), of the structurally and functionally characterized alpha-fucosidase BT_2970 of PUL44 [10], and of a non-PUL beta-galactosidase (*BT_4684*) (**e**). The quantification of sRNA-target binding in panel **d** is based on the mean of three replicate experiments, and the respective point mutations are depicted at the bottom. Source data are provided as a Source Data file.

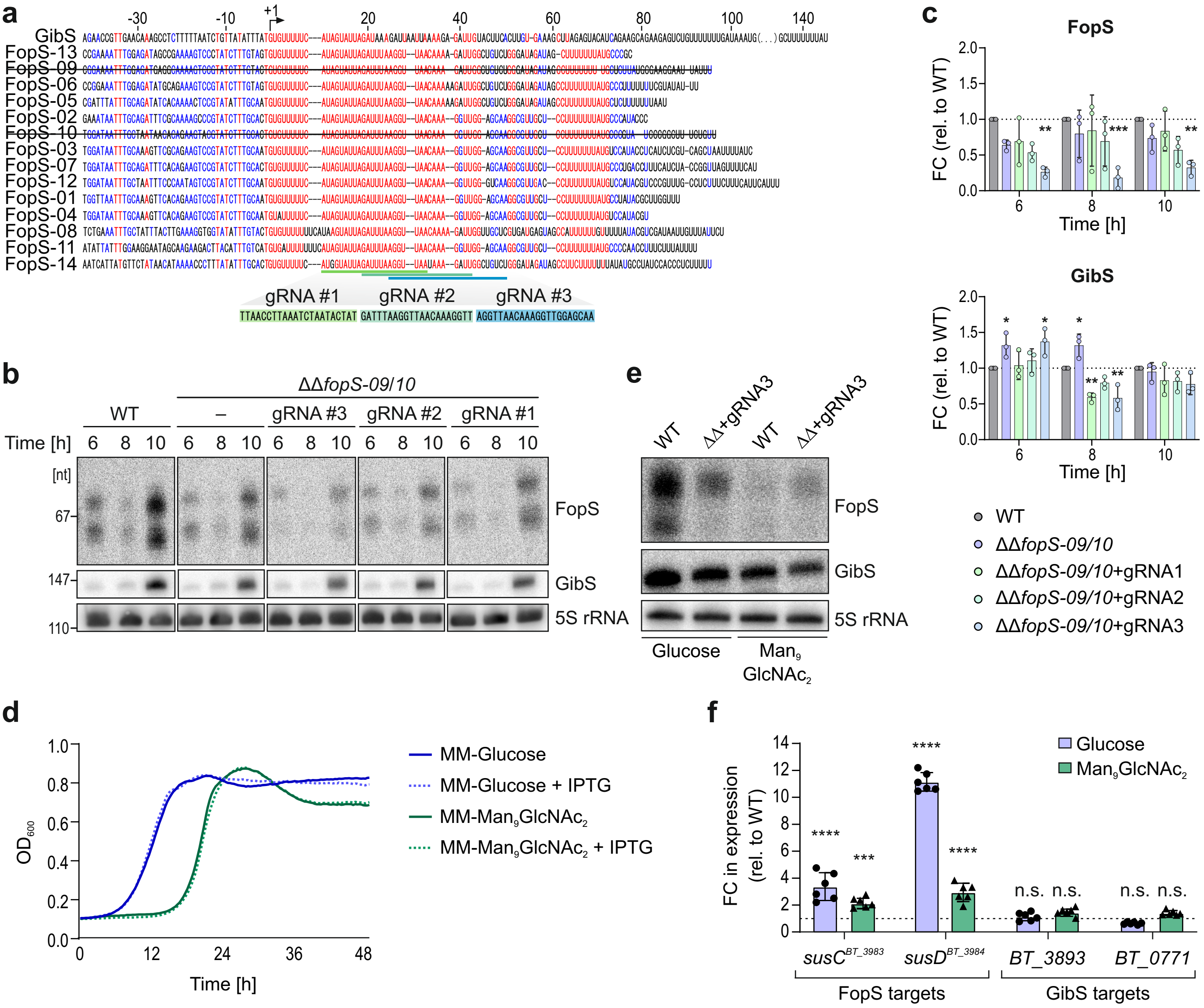

Supplementary Figure 8: Generation and characterization of a multi-*fopS* knockout/knockdown mutant of *B. thetaiotaomicron*. a, Genomic alignment of *fopS* family members with the positions targeted by the three different anti-*fopS* guide RNAs (gRNA #1-3) highlighted. Strikethrough text indicates that CRISPRi was performed in the ΔΔ*fopS-09/10* background. The sequence of *gibS*, which diverges from the *fopS* consensus sequence around the targeted regions, is plotted at the top for comparison. The numbers are relative to the sRNAs' transcriptional start sites (bent arrow). **b,** Representative northern blot experiment to evaluate the knockdown efficiencies of the different gRNAs. The indicated strains were grown in TYG medium containing 250 µM of the CRISPRi inducer IPTG for the indicated time periods, before total RNA was extracted and analyzed. **c,** Quantification of FopS knockdown efficiencies over three biological replicate experiments, one of which is shown in panel **b**. FopS levels were normalized to 5S rRNA abundance and for each time point, the wild-type level was set to 1 (dashed horizontal line). Asterisks: statistical significance was calculated using two-way ANOVA (Sidak's multiple comparisons test; only significant *p*-values [$p < 0.05$] are indicated). **d,** Growth of wild-type *B. thetaiotaomicron* in minimal medium containing either glucose or high-mannose *N*-glycans ($Man_9GlcNAc_2$) as the sole carbon source. Addition of the CRISPRi inducer IPTG (dashed lines) had no effect on bacterial growth kinetics. Lines refer to the mean of two biological replicate experiments, each performed in technical duplicates. **e,** Northern blot of FopS and GibS expression in wild-type *B. thetaiotaomicron* grown for 48 h in glucose- or high-mannose *N*-glycan-containing minimal medium. 5S rRNA was the loading control. **f,** qRT-PCR-based measurement of the steady-state levels of the FopS target operon *susC*$^{BT\_3983}$-*susD*$^{BT\_3984}$ and of established GibS target mRNAs (*BT_3893*, *BT_0771*) in the multi-*fopS* depletion mutant grown in the indicated carbon sources for 48 h, relative to their levels in analogously treated wild-type bacteria (dashed horizontal line). Bars and error bars denote the mean +/-SD of each two biological replicate experiments performed as technical duplicates. Statistical significance was assessed using two-way ANOVA (Sidak's multiple comparisons test; only significant *p*-values [$p < 0.05$] are shown). Note that the magnitude of *susC*$^{BT\_3983}$-*susD*$^{BT\_3984}$ derepression in the *fopS* mutant is larger in glucose as compared to the cognate PUL72 substrate, high-mannose *N*-glycan, reflecting the abundance of FopS sRNAs in wild-type bacteria under those conditions (see panel **e**). Source data are provided as a Source Data file.

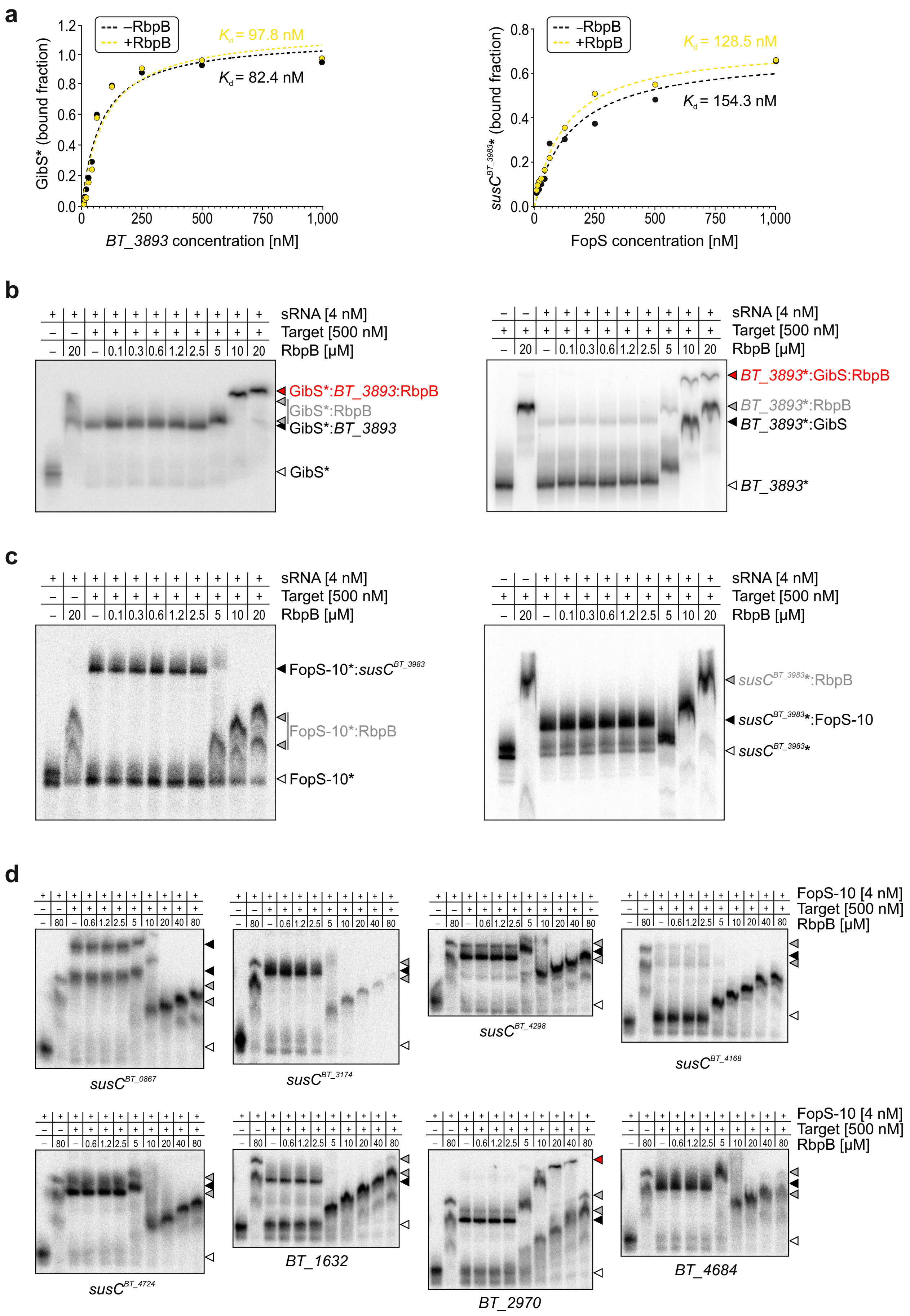
a
–RbpB
+RbpB
K_d = 97.8 nM
K_d = 82.4 nM
GibS* (bound fraction)
BT_3893 concentration [nM]
K_d = 128.5 nM
K_d = 154.3 nM
$susC^{BT\_3983}$* (bound fraction)
FopS concentration [nM]
b
sRNA [4 nM]
Target [500 nM]
RbpB [μM]
GibS*:BT_3893:RbpB
GibS*:RbpB
GibS*:BT_3893
GibS*
BT_3893*:GibS:RbpB
BT_3893*:RbpB
BT_3893*:GibS
BT_3893*
c
FopS-10*:$susC^{BT\_3983}$
FopS-10*:RbpB
FopS-10*
$susC^{BT\_3983}$*:RbpB
$susC^{BT\_3983}$*:FopS-10
$susC^{BT\_3983}$*
d
FopS-10 [4 nM]
Target [500 nM]
RbpB [μM]
$susC^{BT\_0867}$
$susC^{BT\_3174}$
$susC^{BT\_4298}$
$susC^{BT\_4168}$
$susC^{BT\_4724}$
BT_1632
BT_2970
BT_4684

Supplementary Figure 9: *In-vitro* interaction of GibS and FopS with their targets in the presence or absence of RbpB. a, Quantification of EMSAs. K_d values represent the means of three independent replicate experiments performed with either *in vitro*-transcribed and 5' end-labeled GibS or a ~180 nt-long 5' region of *susC*$^{BT\_3983}$ (4 nM each), incubated with increasing concentrations of a 5' segment of *BT_3893* (~150 nt) or FopS-10, respectively. EMSAs were performed in the absence (black line) or presence (yellow line) of 1 µM RbpB. **b,** EMSA reveals the 5' region of *BT_3893* mRNA to be bound by RbpB *in vitro*. In the presence of the GibS sRNA, a ternary complex was formed. **c, d,** Three-component EMSA analogous to panel **b**, but with FopS-10 and its target mRNA, *susC*$^{BT\_3983}$ (**c**), or with radiolabeled FopS-10 and additional, predicted target candidates (**d**). Arrowheads indicate unbound FopS-10 (white), FopS-10:RbpB (grey), FopS-10:target (black), or ternary complexes of FopS-10:RbpB:*BT_2970* (red), respectively. Source data are provided as a Source Data file.

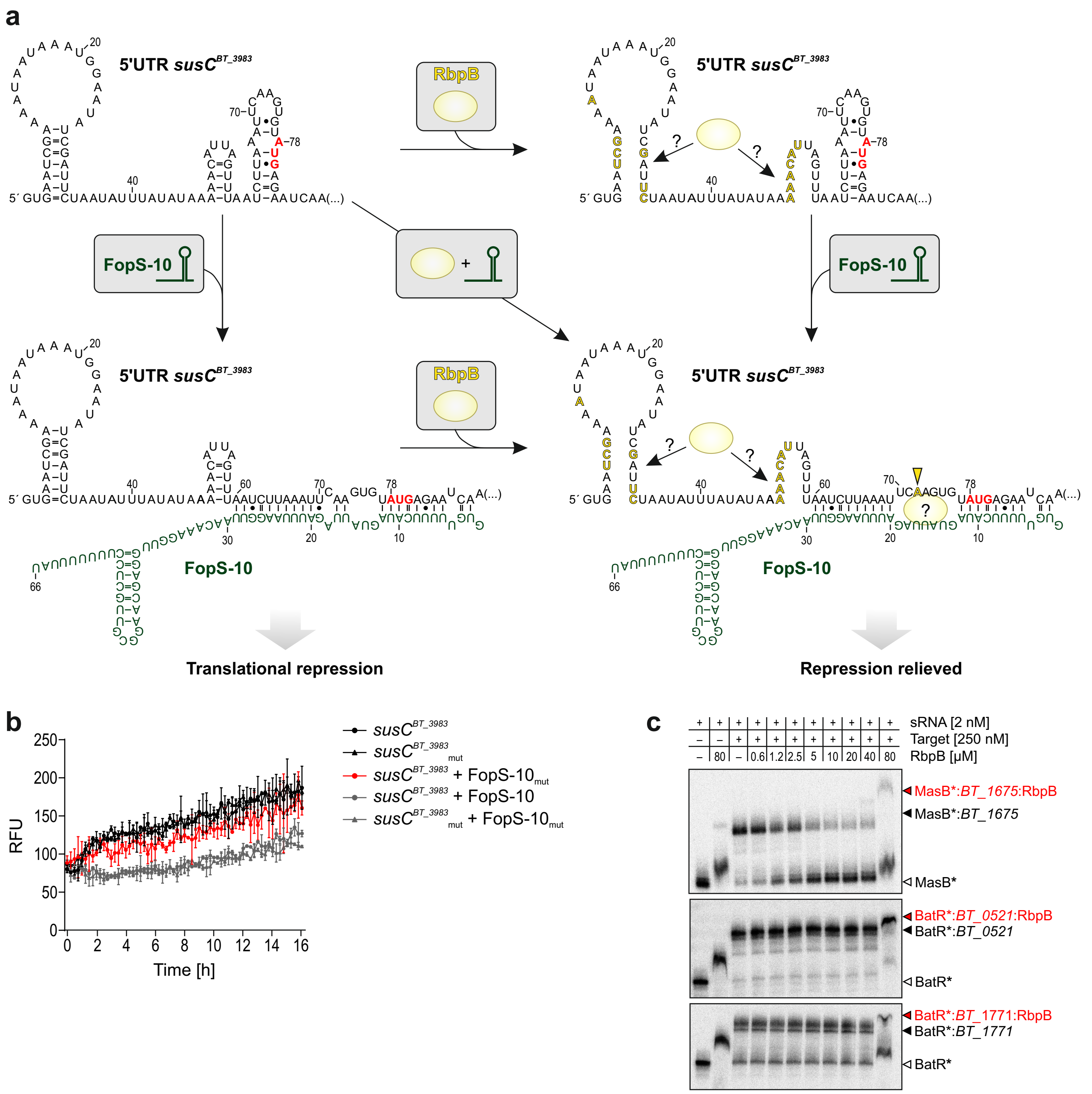

Supplementary Figure 10: Mechanistic insights into the RbpB-mediated relieve of FopS-10 repression and interaction of the protein with other basepairing sRNAs. a, Modelling the interaction of the 5' portion of *susC*$^{BT\_3893}$ with FopS-10, in the presence or absence of RbpB. Intra- and inter-molecular basepair interactions were inferred from the band pattern in the inline probing assay (Fig. 4f). Ribobases involved in rearrangements upon RbpB association (i.e., residues with an altered cleavage efficiency; yellow arrowheads in Fig. 4f) are depicted in yellow font and the translational start codon of the *susC*$^{BT\_3893}$ mRNA in red font. The yellow arrowhead in the lower right schematic highlights the structural change at position –6 relative to the translational start site that occurs specifically when both, FopS-10 and RbpB, are present (enhanced cleavage at position 72 in Fig. 4f). The binding site of RbpB in the 5' single-stranded region of FopS-10 was inferred from CLIP-seq (see Supplementary Fig. 4b). Since *susC*$^{BT\_3893}$ was not expressed in the CLIP-seq condition, the exact binding site of RbpB in this mRNA is unknown; however, the evoked changes in the cleavage pattern in the presence of RbpB could imply that protein binding melts the two 5'-most stem loops (as indicated in the model by arrows with question marks). **b,** *In-vitro* translation assay with different combinations of *5' susC*$^{BT\_3983}$*-gfp-3xFLAG* and FopS-10 (wild-type sequences and point mutations, as introduced in Supplementary Fig. 7d). The assay was analogously performed to the ones described in Fig. 4g, except that the products of the translation reactions were not loaded on a gel, but their accumulation followed over time in a plate reader by measuring the fluorescence intensity emitted by the sample. **c,** Three-component EMSAs to probe the interactions between recombinant RbpB, previously characterized *B. thetaiotaomicron* sRNAs MasB and BatR, and 5' fragments of their respective target mRNAs. Experiments analogous to the ones described in Supplementary Fig. 9b-d. Source data are provided as a Source Data file.

SUPPLEMENTARY REFERENCES

1 Prezza, G. *et al.* Comparative genomics provides structural and functional insights into Bacteroides RNA biology. *Mol Microbiol* **117**, 67-85, doi:10.1111/mmi.14793 (2022).

2 Prezza, G., Liao, C., Reichardt, S., Beisel, C. L. & Westermann, A. J. CRISPR-based screening of small RNA modulators of bile susceptibility in *Bacteroides thetaiotaomicron*. *Proc Natl Acad Sci U S A* **121**, e2311323121, doi:10.1073/pnas.2311323121 (2024).

3 Ryan, D., Jenniches, L., Reichardt, S., Barquist, L. & Westermann, A. J. A high-resolution transcriptome map identifies small RNA regulation of metabolism in the gut microbe Bacteroides thetaiotaomicron. *Nat Commun* **11**, 3557, doi:10.1038/s41467-020-17348-5 (2020).

4 Ryan, D. *et al.* An expanded transcriptome atlas for *Bacteroides thetaiotaomicron* reveals a small RNA that modulates tetracycline sensitivity. *Nat Microbiol* **9**, 1130-1144, doi:10.1038/s41564-024-01642-9 (2024).

5 Fu, L., Niu, B., Zhu, Z., Wu, S. & Li, W. CD-HIT: accelerated for clustering the next-generation sequencing data. *Bioinformatics* **28**, 3150-3152, doi:10.1093/bioinformatics/bts565 (2012).

6 Tisza, M. J. & Buck, C. B. A catalog of tens of thousands of viruses from human metagenomes reveals hidden associations with chronic diseases. *Proc Natl Acad Sci U S A* **118**, doi:10.1073/pnas.2023202118 (2021).

7 Martens, E. C., Chiang, H. C. & Gordon, J. I. Mucosal glycan foraging enhances fitness and transmission of a saccharolytic human gut bacterial symbiont. *Cell Host Microbe* **4**, 447-457, doi:10.1016/j.chom.2008.09.007 (2008).

8 Martens, E. C. *et al.* Recognition and degradation of plant cell wall polysaccharides by two human gut symbionts. *PLoS Biol* **9**, e1001221, doi:10.1371/journal.pbio.1001221 (2011).

9 Luis, A. S. *et al.* Dietary pectic glycans are degraded by coordinated enzyme pathways in human colonic Bacteroides. *Nat Microbiol* **3**, 210-219, doi:10.1038/s41564-017-0079-1 (2018).

10 Wright, D. W., Moreno-Vargas, A. J., Carmona, A. T., Robina, I. & Davies, G. J. Three dimensional structure of a bacterial α-l-fucosidase with a 5-membered iminocyclitol inhibitor. *Bioorg Med Chem* **21**, 4751-4754, doi:10.1016/j.bmc.2013.05.056 (2013).
