# Supplementary material for: The global RNA-binding protein RbpB is a regulator of polysaccharide utilization in Bacteroides thetaiotaomicron
Source: Nat Commun. 2025 Jan 2;16:208. doi: 10.1038/s41467-024-55383-8 (PMC11697453; doi:10.1038/s41467-024-55383-8)
Supplement: Supplementary file 2 — Description of Additional Supplementary Files [file 41467_2024_55383_MOESM2_ESM.docx]

**SUPPLEMENTARY DATA FILE CAPTIONS**

**Supplementary Data 1: Combined CLIP-seq data.** Statistically significant peaks (*p*_adj_ ≤ 0.05, Benjamini-Hochberg corrected; log_2_ FC > 2) are listed. This table forms the basis for the annotation of RbpB binding sites in the *B*. *thetaiotaomicron* transcriptome, as is accessible at https://bacteroides.helmholtz-hzi.de/jbrowse_new/.

**Supplementary Data 2: IntaRNA prediction of FopS targets.** IntaRNA 2.0 was run with default settings, including default statistical parameters (FDR, *p* value). The table features the 100 top-scoring target candidates. Targets experimentally validated by EMSA (see Supplementary Fig. 7e) are in red.

**Supplementary Data 3: List of bacterial strains, plasmids, antibodies, and oligonucleotides used in this study.**
