# Supplementary material for: The global RNA-binding protein RbpB is a regulator of polysaccharide utilization in Bacteroides thetaiotaomicron
Source: Nat Commun. 2025 Jan 2;16:208. doi: 10.1038/s41467-024-55383-8 (PMC11697453; doi:10.1038/s41467-024-55383-8)
Supplement: Supplementary file 6 — Reporting Summary [file 41467_2024_55383_MOESM6_ESM.pdf]

nature portfolio

Corresponding author(s): Alexander J. Westermann

Last updated by author(s): Nov 21, 2024

# Reporting Summary

Nature Portfolio wishes to improve the reproducibility of the work that we publish. This form provides structure for consistency and transparency in reporting. For further information on Nature Portfolio policies, see our Editorial Policies and the Editorial Policy Checklist.

## Statistics

For all statistical analyses, confirm that the following items are present in the figure legend, table legend, main text, or Methods section.

| n/a | Confirmed | |
|---|---|---|
| ☐ | ☒ | The exact sample size (*n*) for each experimental group/condition, given as a discrete number and unit of measurement |
| ☒ | ☐ | A statement on whether measurements were taken from distinct samples or whether the same sample was measured repeatedly |
| ☐ | ☒ | The statistical test(s) used AND whether they are one- or two-sided<br>*Only common tests should be described solely by name; describe more complex techniques in the Methods section.* |
| ☒ | ☐ | A description of all covariates tested |
| ☐ | ☒ | A description of any assumptions or corrections, such as tests of normality and adjustment for multiple comparisons |
| ☐ | ☒ | A full description of the statistical parameters including central tendency (e.g. means) or other basic estimates (e.g. regression coefficient) AND variation (e.g. standard deviation) or associated estimates of uncertainty (e.g. confidence intervals) |
| ☐ | ☒ | For null hypothesis testing, the test statistic (e.g. *F*, *t*, *r*) with confidence intervals, effect sizes, degrees of freedom and *P* value noted<br>*Give P values as exact values whenever suitable.* |
| ☒ | ☐ | For Bayesian analysis, information on the choice of priors and Markov chain Monte Carlo settings |
| ☒ | ☐ | For hierarchical and complex designs, identification of the appropriate level for tests and full reporting of outcomes |
| ☐ | ☒ | Estimates of effect sizes (e.g. Cohen's *d*, Pearson's *r*), indicating how they were calculated |

*Our web collection on statistics for biologists contains articles on many of the points above.*

## Software and code

Policy information about availability of computer code

| | |
|---|---|
| Data collection | NextSeq 500 platform (Ilumina) |
| Data analysis | Filtering and trimming  raw reads:BBDuk<br>Read De-duplication:FastUniq 1.1<br>Mapping: READemption 1.0.5 pipeline using segemehl 0.3.4<br>Peak calling: PEAKachu pipeline version 0.2.0 (https://github.com/tbischler/PEAKachu)<br>Pathway enrichement analysis: enricher function of the clusterProfiler R package version 3.14.3<br>CLIP-seq normalization: (https://github.com/lbarquist/norclip)<br>In-silico interaction prediction: IntaRNA version  3.3.1 linking Vienna RNA package 2.5.0<br>16S rRNA sequencing analysis: Qiime2-amplicon-2024.5 |

For manuscripts utilizing custom algorithms or software that are central to the research but not yet described in published literature, software must be made available to editors and reviewers. We strongly encourage code deposition in a community repository (e.g. GitHub). See the Nature Portfolio guidelines for submitting code & software for further information.

## Data

Policy information about availability of data

All manuscripts must include a data availability statement. This statement should provide the following information, where applicable:

- Accession codes, unique identifiers, or web links for publicly available datasets
- A description of any restrictions on data availability
- For clinical datasets or third party data, please ensure that the statement adheres to our policy

Sequencing data are available at NCBI Gene Expression Omnibus under the accession number GSE244816 (https://www.ncbi.nlm.nih.gov/geo/query/acc.cgi?acc=GSE244816). Source data are provided with this paper.

## Research involving human participants, their data, or biological material

Policy information about studies with human participants or human data. See also policy information about sex, gender (identity/presentation), and sexual orientation and race, ethnicity and racism.

| | |
|---|---|
| Reporting on sex and gender | N/A |
| Reporting on race, ethnicity, or other socially relevant groupings | N/A |
| Population characteristics | N/A |
| Recruitment | N/A |
| Ethics oversight | N/A |

Note that full information on the approval of the study protocol must also be provided in the manuscript.

# Field-specific reporting

Please select the one below that is the best fit for your research. If you are not sure, read the appropriate sections before making your selection.

☒ Life sciences ☐ Behavioural & social sciences ☐ Ecological, evolutionary & environmental sciences

For a reference copy of the document with all sections, see nature.com/documents/nr-reporting-summary-flat.pdf

# Life sciences study design

All studies must disclose on these points even when the disclosure is negative.

| | |
|---|---|
| Sample size | Mouse experiments were performed in two independent experiments, each comprised of four biological replicates. CLIP-seq was performed in biological duplicates. Northern blotting, EMSAs in-line probing and in vitro translations were all performed in two independent experiments. qRT-PCR assay were performed in three biological replicates, each consisting of two technical replicates. No statistical methods were used to predetermine sample size. Sample sizes were selected based on our prior experience with similar experimental setups and similar experiments descibed in published papers. |
| Data exclusions | No data were excluded |
| Replication | Mouse experiments were performed in two independent experiments, each comprised of four biological replicates. CLIP-seq was performed in biological duplicates. Northern blotting, EMSAs in-line probing and in vitro translations were all performed in two independent experiments. qRT-PCR assay were performed in three biological replicates, each consisting of two technical replicates. All sample sizes were selected based on prior experience with similar experimental setups. |
| Randomization | Animals were randomly assigned into treatment groups (cages) prior to any experiment. |
| Blinding | Sample blinding was not relevant as none of the readouts was subjective. |

# Reporting for specific materials, systems and methods

We require information from authors about some types of materials, experimental systems and methods used in many studies. Here, indicate whether each material, system or method listed is relevant to your study. If you are not sure if a list item applies to your research, read the appropriate section before selecting a response.

## Materials & experimental systems

| n/a | Involved in the study | |
|---|---|---|
| ☐ | ☒ | Antibodies |
| ☒ | ☐ | Eukaryotic cell lines |
| ☒ | ☐ | Palaeontology and archaeology |
| ☐ | ☒ | Animals and other organisms |
| ☒ | ☐ | Clinical data |
| ☒ | ☐ | Dual use research of concern |
| ☒ | ☐ | Plants |

## Methods

| n/a | Involved in the study | |
|---|---|---|
| ☒ | ☐ | ChIP-seq |
| ☒ | ☐ | Flow cytometry |
| ☒ | ☐ | MRI-based neuroimaging |

# Antibodies

Antibodies used

For western blot experiments, we used a primary antibody against FLAG (Mouse, Sigma-Aldrich, cat# F1804, 1:1,000 dilution)
As secondary antibody, we used goat anti-mouse IgG, HRP (Goat, ThermoFisherScientific, cat# 31430, 1:10,000 dilution)

Validation

Validation data and citations of the primary antibody can be found on the manufacturer's website: https://www.sigmaaldrich.com/deepweb/assets/sigmaaldrich/product/documents/119/160/f1804bul-mk.pdf.

# Animals and other research organisms

Policy information about studies involving animals; ARRIVE guidelines recommended for reporting animal research, and Sex and Gender in Research

Laboratory animals

Mouse strain: C57BL/6J wild-type (cat# 000664), Age: seven to nine-week-old
sex: female or male mice were randomly assigned into treatment groups before the experiment.
C57BL/6J wild-type (cat# 000664), were obtained from Jackson Laboratory. Mice were housed in sterile cages under specific pathogen-free conditions on a 12-hour light cycle, with ad libitum access to food and sterile water at Vanderbilt University Medical Center. Seven to nine-week-old female or male mice were randomly assigned into treatment groups before the experiment. One week before antibiotic treatment, mice were switched to a low fiber diet (TD.86489) or remained on a control diet 5010 (LabDiet 0001326) until the end of the experiment. Antibiotic cocktails (ampicillin [Sigma-Aldrich], metronidazole [Sigma-Aldrich], vancomycin [Chem Impex International], and neomycin [Sigma-Aldrich]; 5 mg of each per mouse) were administered by oral gavage daily for 5 days. After antibiotic treatment, mice were inoculated with an equal mixture of 0.5 x 109 CFU of the B. thetaiotaomicron wild-type strain and 0.5 x 109 CFU of the ΔrbpB mutant for 6 days. After euthanasia, cecal and colonic tissue was collected in sterile PBS, and the abundance of B. thetaiotaomicron strains was quantified by plating serial-diluted intestinal contents on selective agar.

Wild animals

Study did not involve wild animals.

Reporting on sex

We observed a sex-specific difference, in which the phenotype was more prominent in males than in females.

Field-collected samples

Study did not involve samples collected from the field.

Ethics oversight

All experiments were conducted in accordance with the policies of the Institutional Animal Care and Use Committee at Vanderbilt University Medical Center (protocol # M2300019-00).

Note that full information on the approval of the study protocol must also be provided in the manuscript.

# Plants

Seed stocks

*Report on the source of all seed stocks or other plant material used. If applicable, state the seed stock centre and catalogue number. If plant specimens were collected from the field, describe the collection location, date and sampling procedures.*

Novel plant genotypes

*Describe the methods by which all novel plant genotypes were produced. This includes those generated by transgenic approaches, gene editing, chemical/radiation-based mutagenesis and hybridization. For transgenic lines, describe the transformation method, the number of independent lines analyzed and the generation upon which experiments were performed. For gene-edited lines, describe the editor used, the endogenous sequence targeted for editing, the targeting guide RNA sequence (if applicable) and how the editor was applied.*

Authentication

*Describe any authentication procedures for each seed stock used or novel genotype generated. Describe any experiments used to assess the effect of a mutation and, where applicable, how potential secondary effects (e.g. second site T-DNA insertions, mosiacism, off-target gene editing) were examined.*
